# TFAM‐Mediated mtDNA Replication is Essential for Developmental Competence of In Vitro Grown Oocytes

**DOI:** 10.1002/rmb2.70031

**Published:** 2026-02-23

**Authors:** Son Quang Do, Hidetaka Tasaki, Hiroaki Funahashi, Takuya Wakai

**Affiliations:** ^1^ Department of Animal Science, Graduate School of Environmental, Life, Natural Science and Technology Okayama University Okayama Japan; ^2^ Assisted Reproductive Technology Center Okayama University Okayama Japan

**Keywords:** in vitro growth, mitochondrial biogenesis, mtDNA, oogenesis, TFAM

## Abstract

**Purpose:**

Mitochondria are essential for oocyte maturation and early embryonic development, supplying ATP and maintaining mitochondrial DNA (mtDNA) integrity. During oogenesis, mtDNA undergoes dramatic amplification, but the mechanisms and functional significance of this process remain unclear. The purpose of this study was to elucidate the role of mitochondrial transcription factor A (TFAM) in mouse oocytes using an in vitro growth (IVG) system.

**Methods:**

Oocytes at different growth stages were analyzed for mtDNA copy number and expression of mitochondrial biogenesis genes. To assess TFAM function, siRNA targeting *Tfam* was microinjected into secondary follicles, which were then cultured for 12 days under IVG conditions. Following culture, oocyte growth, mtDNA content, mitochondrial membrane potential, and developmental competence after in vitro fertilization (IVF) were evaluated.

**Results:**

mtDNA copy number increased nonlinearly during oocyte growth, with a pronounced rise at the secondary follicle stage accompanied by TFAM upregulation. TFAM knockdown reduced mtDNA copy number and mitochondrial function without affecting oocyte size or meiotic maturation, but significantly decreased blastocyst formation and total cell numbers per blastocyst.

**Conclusions:**

TFAM‐mediated mtDNA replication is crucial for mitochondrial function and developmental competence of IVG‐derived oocytes, underscoring its importance in early embryonic development.

## 1 Introduction

Oogenesis is a highly coordinated developmental process that underlies female fertility. In mice, primordial germ cells (PGCs) are specified around embryonic day 7.25 (E7.25) and proliferate until approximately E13.5, when they enter meiotic prophase I before birth [1]. After birth, oocytes become enclosed by somatic cells to form primordial follicles [2]. During follicular growth, the oocyte expands nearly 300‐fold in volume while maintaining constant communication with surrounding granulosa cells via gap junctions. This growth phase is accompanied by the accumulation of maternal mRNA and the generation of cytoplasmic organelles, which together establish the foundation for a functional oocyte.

Among these maternal components, mitochondria are particularly critical. As semi‐autonomous organelles containing their own genome (mtDNA), mitochondria undergo extensive replication during oocyte growth, resulting in mature oocytes that contain several hundred thousand copies of mtDNA [3]. This dramatic amplification ensures a sufficient mitochondrial content for subsequent embryogenesis, because mtDNA replication remains quiescent from fertilization until the blastocyst stage [4]. Consequently, early embryonic development depends on the mitochondrial pool established during oogenesis [5]. In addition to transmitting mtDNA across generations, oocyte mitochondria provide ATP through oxidative phosphorylation to support fertilization and preimplantation development [6]. Mitochondrial dysfunction in oocytes has been associated with aging [7], obesity [8], diabetes [9], all of which increase the risk of infertility.

Within mitochondria, mtDNA is packaged into nucleoids along the inner membrane [10]. Mitochondrial transcription factor A (TFAM), a nuclear‐encoded protein, is a core component of the mitochondrial nucleoid and is abundantly present in mitochondria, where it packages mtDNA in a histone‐like manner [11, 12]. Through its DNA‐binding properties, TFAM regulates mtDNA replication and transcription by stabilizing, bending, and unwinding the mitochondrial genome, as well as by initiating transcription at promoter regions [13]. Maintaining an optimal TFAM‐to‐mtDNA ratio is essential for mitochondrial homeostasis, as both TFAM depletion [14, 15, 16] and overexpression [14, 17] disrupt mtDNA replication and reduce mtDNA copy number. Despite its central role, the physiological function of TFAM during oocyte growth and its contribution to mtDNA regulation and oocyte competence remain poorly understood.

Although high mtDNA abundance is a hallmark of oogenesis, the amount required for developmental competence remains debated. Oocytes exhibit substantial variation in mtDNA copy numbers, suggesting the existence of a flexible threshold. Notably, oocyte‐specific depletion of TFAM markedly reduces mtDNA content; yet oocytes containing as few as ~4,000 mtDNA copies can still reach the blastocyst stage, and successful postimplantation development has been reported with approximately 50 000 copies of mtDNA [18]. These findings raise the question of why oocytes normally accumulate such a large mitochondrial pool and support the idea that mtDNA amplification serves as an energetic reserve to ensure adequate ATP production during early development.

Importantly, the threshold level of mtDNA required to sustain embryonic development is likely to differ between in vivo and in vitro environments. The in vivo follicular environment provides complex and dynamic support through gap junction–mediated communication [19, 20], metabolic coupling [21], and paracrine signaling [22, 23], which are not fully recapitulated under in vitro culture conditions. Bidirectional communication between the oocyte and surrounding cumulus and granulosa cells is more robust in vivo and plays a critical role in supplying metabolites, maintaining redox balance, and supporting mitochondrial metabolism and quality control. In contrast, these regulatory interactions are suboptimal under in vitro culture conditions [24]. Consistent with this notion, reduced mtDNA copy number correlates with poor oocyte quality and developmental failure in in vitro systems [25, 26], whereas enhanced mtDNA replication improves developmental outcomes [25, 27].

Recent advances in in vitro culture for ovaries, follicles, and granulosa cell–oocyte complexes have enabled detailed study of mammalian oogenesis and the preservation of genetic resources in livestock, endangered species, and humans. However, oocytes derived from in vitro growth (IVG) systems exhibit reduced developmental competence, compared with in vivo–grown oocytes. This impairment is characterized by decreased mitochondrial membrane potential (MMP) and ATP production, increased reactive oxygen species (ROS) levels, and abnormal mitochondrial distribution [28] These observations suggest that impaired mitochondrial function and metabolic buffering may underlie the reduced competence of IVG oocytes, and the lower threshold of mtDNA numbers for normal development in IVG oocytes may be different from in vivo derived oocytes [18].

To address this possibility, we analyzed the expression of mitochondrial regulatory genes during oocyte growth in an IVG system and investigated the functional importance of TFAM in mtDNA replication and oocyte developmental competence using TFAM knockdown experiments. Our study aimed to clarify how TFAM‐mediated mtDNA replication contributes to mitochondrial quantity, mitochondrial quality, and developmental competence in IVG oocytes.

## 2 Materials and Methods

### 2.1 Isolation of Oocytes for mtDNA and RNA Extraction

All mice were housed in a pathogen‐free, temperature and humidity‐controlled environment on 12‐h light/12‐h dark cycles in filter‐top cages and fed a standard diet *ad libitum*. B6DBF1 mice used in this study were generated in‐house from crossing of female C57BL/6N with male DBA/2. Animal experiments were conducted according to the guidelines of the Animal Care and Use Committee of Okayama University under approval number OKU–2019173.

Ovaries were collected from BDF1 mice at 7–12 days postnatal. The tissues were digested with 0.1% (w/v) collagenase type I, followed by 0.05% Trypsin–EDTA to obtain denuded growing oocytes. Mature female mice (> 8 weeks old) were primed with 7.5 IU pregnant mare serum gonadotropin (PMSG, ASKA Pharmaceutical, Tokyo, Japan). After 42–46 h, oocyte–cumulus complexes were isolated by mechanical puncture, and cumulus cells were removed by gentle pipetting to obtain denuded, fully grown oocytes. Oocyte diameters were measured and categorized according to previously described criteria [29].

For mtDNA quantification, individual oocytes were washed in PBS containing 0.1% (w/v) polyvinyl alcohol (PVA, Thermo Fisher Scientific, Massachusetts, USA), transferred into 1 μL of buffer in individual wells of an 8‐well strip tube, snap‐frozen in liquid nitrogen, and stored at −30°C until DNA extraction. For total RNA extraction, pools of 50 denuded oocytes were washed in PBS/PVA, transferred into a 1.5 mL microcentrifuge tube containing 10 μL RNAlater (Thermo Fisher Scientific, Massachusetts, USA), snap‐frozen, and stored at −80°C until use.

### 2.2 Quantification of mtDNA Copy Number and Gene Expression

Total DNA was extracted by adding 9 μL of lysis buffer (50 mM Tris–HCl, 0.5% Tween‐20, 0.1 mg/mL proteinase K) to each tube containing a single oocyte. Samples were incubated at 55°C for 30 min and heat‐inactivated at 95°C for 10 min. Absolute quantification standards were generated from a fragment of the ND1 gene cloned into a pTAC2 plasmid (BioDynamics Laboratory Inc., Tokyo, Japan), treated with T5 exonuclease (New England Biolabs, Massachusetts, USA), and linearized with BamHI, as previously described [30, 31].

Total RNA was isolated using TRIzol reagent (Thermo Fisher Scientific, Massachusetts, USA) and reverse‐transcribed from 100 ng total RNA using the High‐Capacity cDNA Reverse Transcription Kit (Thermo Fisher Scientific, Massachusetts, USA). Real‐time PCR was performed using a LightCycler 96 system (Roche, Basel, Switzerland) with GeneAce SYBR qPCR Mix α (Nippon Gene, Tokyo, Japan) in a 10 μL reaction containing 2 μL of DNA or cDNA and 0.5 μM of each primer (Table S1). The thermal profile consisted of 95°C for 10 min, followed by 40 cycles of 95°C for 15 s and 60°C for 60 s. Melt curve analysis was conducted from 65°C to 95°C with continuous fluorescence acquisition.

Absolute mtDNA copy numbers were determined from a six‐point, 10‐fold serial dilution standard curve (10^7^–10^2^ copies). Relative gene expression was normalized to reference genes (*Ppia*, *H2afz, Actb*) using the 2^−ΔΔCt^ method. All reactions were performed in duplicate.

### 2.3 Isolation, Microinjection, and Culture of Secondary Follicles

Secondary follicles were isolated and cultured as previously described [32]. Ovaries were collected from 10 to 12‐day‐old mice, and follicles (100–130 μm in diameter) were selected for culture. Microinjection was performed as previously described [33, 34]. Briefly, follicle‐enclosed oocytes were injected with 2.5–5 pL of 2 μM DsiRNA targeting *Tfam* (mm.Ri.Tfam.13.1, IDT, Iowa, USA) or 2 μM negative control DsiRNA (51–01–14‐03, IDT, Iowa, USA) directly into the ooplasm.

Follicles were cultured on Millicell culture inserts (PICM0RG50, Merck, New Jersey, USA) in αMEM (Gibco, Massachusetts, USA) supplemented with 1.5 mM ascorbic acid, 2% (w/v) polyvinylpyrrolidone‐360, 5% (v/v) FBS (Gibco, Massachusetts, USA), and 0.1 IU/mL FSH (Follistim Follitropin Injection 300, Organon, New Jersey, USA). Up to 50 follicles were cultured at 37°C in a humidified atmosphere of 5% CO_2_ for 12 days, with half of the medium replaced every 2 days. After culture, oocyte–cumulus complexes (OCCs) were collected for further analysis.

### 2.4 In Vitro Maturation, In Vitro Fertilization, and in Vitro Embryo Culture

For in vitro maturation (IVM), OCCs were cultured in αMEM supplemented with 1.5 mM ascorbic acid, 5% (v/v) FBS, 0.1 IU/mL FSH, 4 ng/mL EGF, and 1.2 IU/mL hCG. Up to 40 OCCs were cultured in 400 μL IVM medium at 37°C for 17 h in 5% CO_2_.

For in vitro fertilization (IVF), cumulus–oocyte complexes with expanded cumulus were transferred to human tubal fluid (HTF) medium and inseminated with capacitated sperm (1 × 10^5^ sperm/mL) for 6 h at 37°C in 5% CO_2_. After IVF, cumulus cells were removed by gentle pipetting, and zygotes with two pronuclei were cultured in KSOM medium for 4.5 days under identical conditions. Embryos displaying a defined blastocoel were classified as blastocysts. Blastocysts were fixed with 4% paraformaldehyde (PFA) in PBS, stained with Hoechst 33244, and imaged under a fluorescence microscope to count cell numbers.

### 2.5 Immunofluorescence

Oocytes were fixed with 4% PFA in PBS, permeabilized with 0.1% Triton X‐100 (Thermo Fisher, USA) for 30 min at room temperature, and blocked with 1% BSA in PBS for 24 h at 4°C. Samples were incubated overnight at 4°C with primary antibodies against α‐tubulin (11224–1‐AP, ProteinTech, Illinois, USA; 1:200) or TFAM (ab131607, Abcam, Cambridge, UK; 1:400), followed by incubation with Alexa Fluor 594–conjugated secondary antibody (A‐11012, Invitrogen, Massachusetts, USA; 1:500) for 1 h at 37°C. Nuclei were counterstained with DAPI. Images were captured using an inverted fluorescence microscope (BZ‐X810, Keyence, Osaka, Japan) or a confocal laser‐scanning microscope (FV‐1200, Olympus, Tokyo Japan), and fluorescence intensity was quantified using ImageJ software v1.53k (National Institutes of Health, USA).

### 2.6 Measurement of MMP, Mitochondrial Content, and ROS Production

Freshly denuded oocytes were incubated in M2/IMBX medium containing 25 nM tetramethylrhodamine methyl ester perchlorate (TMRM; Sigma‐Aldrich, Missouri, USA) and 10 μM CM‐H₂DCFDA (Thermo Fisher, Massachusetts, USA) at 37°C for 30 min. One‐cell embryos after IVF were incubated in KSOM medium containing 1 μM MitoTracker Green FM (Thermo Fisher, Massachusetts, USA) and 25 nM TMRM. Oocytes/embryos were washed and imaged under identical exposure conditions using a fluorescence microscope. Fluorescence intensities were quantified using ImageJ software v1.53k.

### 2.7 Statistical Analysis

Statistical analyses were performed using GraphPad Prism 7 (GraphPad Software, California, USA). Regression analysis was conducted using polynomial quadratic regression. Percentage data were normalized by arc‐sine transformation before analysis. When significant differences were detected, Bonferroni/Dunn post hoc tests were applied. Each experiment was independently repeated at least three times. Data are presented as mean ± SEM, and statistical significance was defined as *p* < 0.05.

## 3 Results

### 3.1 Changes in mtDNA Copy Number During Oocyte Growth

To investigate the dynamics of mtDNA accumulation during oocyte growth, oocytes were isolated from mice at different developmental stages, individually collected, and measured for diameter prior to mtDNA quantification. Because mammalian oogenesis proceeds in overlapping cohorts [35], follicles within a single ovary represent a range of developmental stages. Based on oocyte diameter [29], oocytes were collected from follicles classified as follows: primary follicle from 8‐day‐old mice, secondary/preantral to antral follicles from 12‐day‐old mice, and early antral to Graafian follicles from adult mice.

**FIGURE 1:**
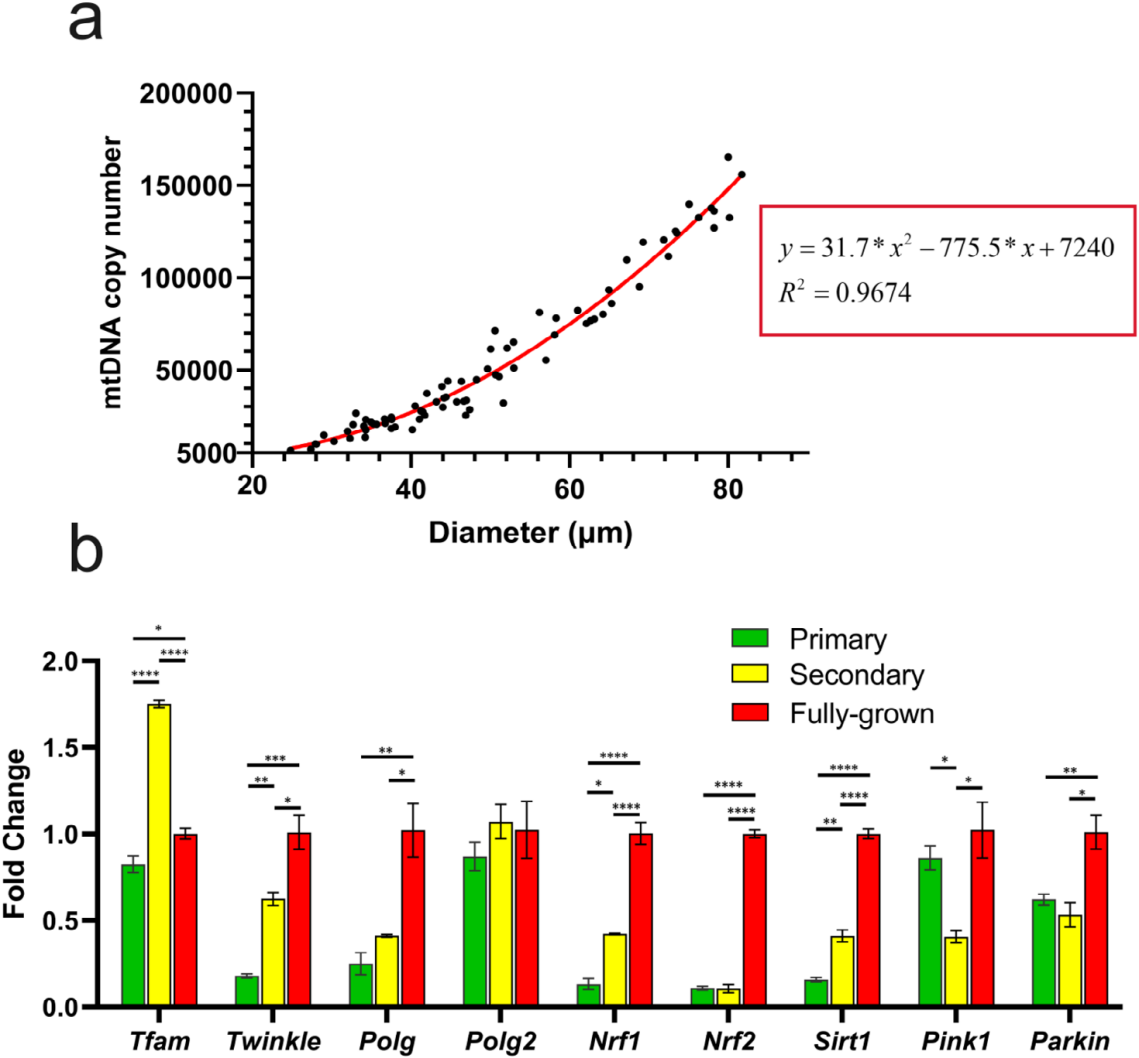
Mitochondrial DNA replication dynamics during mouse oocyte growth. (a) Relationship between mtDNA copy number and oocyte diameter. Data were fitted using a polynomial quadratic regression (red line). The inset boxes show the regression equation and *R* ^2^ value. (b) Relative expression of genes involved in mitochondrial biogenesis and mtDNA replication at different oocyte growth stages. Expression levels are shown as fold changes relative to fully grown oocytes. Data represent mean ± SEM from three independent experiments. Asterisks indicate statistical significance (*p < 0.05; **p < 0.01; ***p < 0.001; ****p < 0.0001).

mtDNA copy number increased steadily during oocyte growth, from approximately 5000 copies in early‐stage oocytes to ~160,000 copies in fully grown oocytes (Figure 1a). Polynomial quadratic regression analysis revealed an excellent fit (R^2^ = 0.9674), indicating that mtDNA accumulation during oocyte growth follows a non‐linear pattern that accelerates at later growth stages.

### 3.2 Changes in Mitochondrial Regulatory Gene Expression During Oocyte Growth

To explore the molecular basis underlying the non‐linear increase in mtDNA, we examined the expression of genes associated with mitochondrial biogenesis, mtDNA replication, and mitochondrial quality control in primary, secondary, and fully grown oocytes. Transcripts of *Twinkle*, *Polg*, *Nrf1*, *Nrf2*, *Sirt1*, and *Parkin* progressively increased during oocyte growth, with marked upregulation at later stages. Notably, *Tfam* mRNA exhibited a sharp increase at the secondary follicle stage, whereas *Pink1*, a key regulator of mitophagy, was downregulated at this stage (Figure 1b). These findings indicate that mitochondrial biogenesis intensifies as oocytes mature, and that the secondary follicle stage represents a critical transition point characterized by enhanced mtDNA replication and reduced mitochondrial turnover.

### 3.3 TFAM Knockdown Reduces mtDNA Copy Number and Mitochondrial Membrane Potential

Because TFAM is a central regulator of mtDNA replication and transcription, we next examined its role in oogenesis using RNA interference. Secondary follicles were microinjected with siRNA targeting *Tfam* (siTFAM groups) or non‐targeting siRNA (siNTC groups) and cultured under IVG conditions.

**FIGURE 2:**
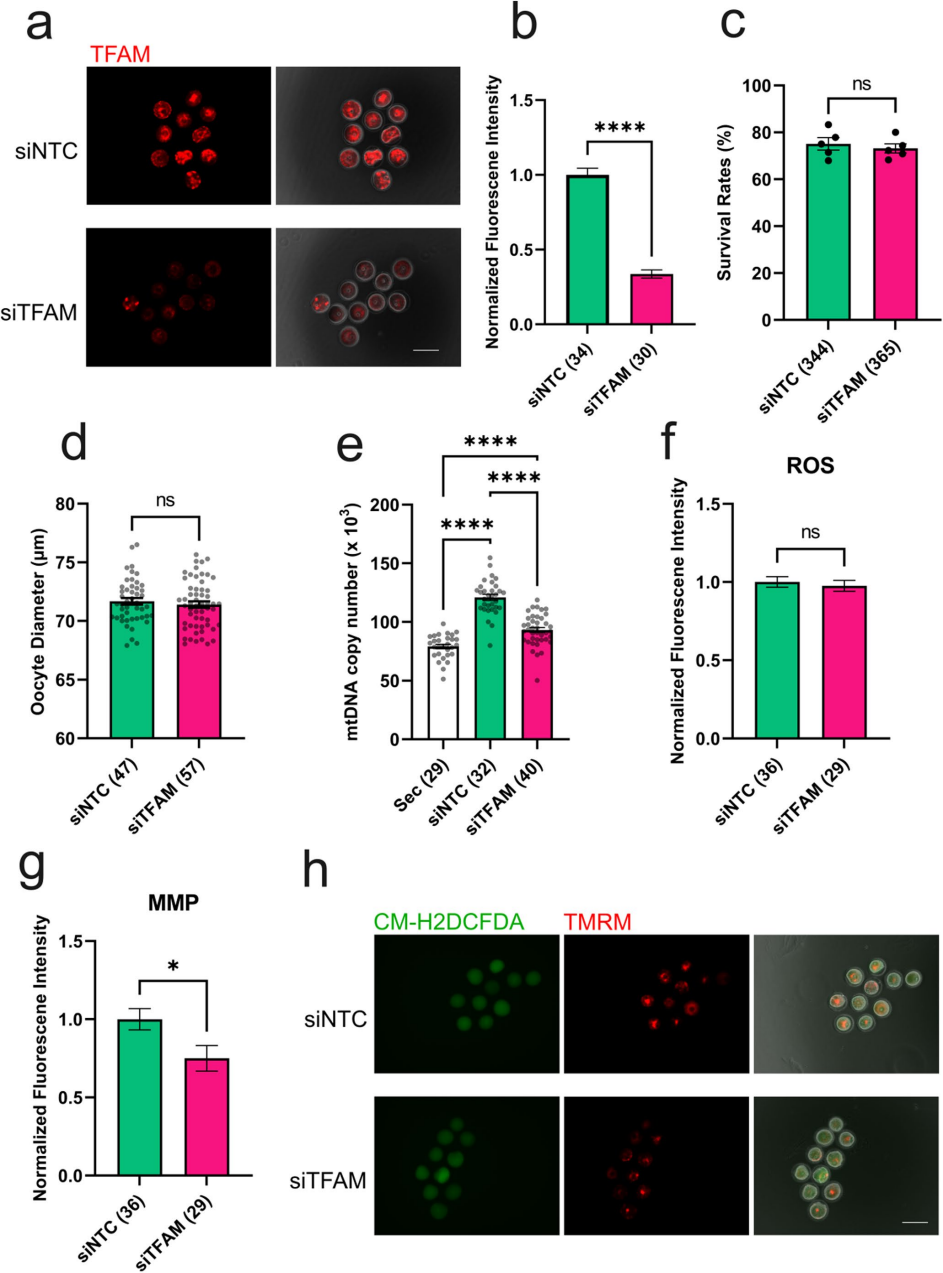
TFAM is required for mitochondrial biogenesis and function during oocyte growth. (a) Representative immunofluorescence images showing TFAM expression in control (siNTC) and *Tfam* knockdown (siTFAM) oocytes. (b) Quantification of TFAM fluorescence intensity. (c) Follicle survival rate after 12 days of in vitro growth (IVG). (d) Oocyte diameters after IVG. (e) mtDNA copy numbers in control and TFAM knockdown oocytes. (f) Normalized intracellular reactive oxygen species (ROS) levels. (g) Normalized mitochondrial membrane potential (MMP) levels. (h) Representative fluorescence images showing ROS and MMP signals in control and TFAM knockdown oocytes. Data represent mean ± SEM from three independent experiments. Asterisks indicate statistical significance (*p < 0.05; ****p < 0.0001). Scale bars, 100 μm. siNTC, non‐targeting siRNA; siTFAM, *Tfam*‐targeting siRNA.

Immunofluorescence and qPCR confirmed efficient TFAM knockdown in oocytes (Figure 2a,b, Figure S1). After 12 days of IVG, TFAM depletion had no apparent effect on follicle survival (Figure 2c) or oocyte size (Figure 2d). However, TFAM knockdown significantly (*p* < 0.0001) reduced mtDNA copy number (Figure 2e).

To assess mitochondrial function, we measured ROS levels and MMP. TFAM depletion did not significantly alter ROS levels (Figure 2f,g) but significantly (*p* < 0.05) decreased MMP (Figure 2h). These results demonstrate that TFAM is essential for proper mtDNA amplification and maintenance of mitochondrial functional integrity during oocyte growth.

### 3.4 TFAM Knockdown Impairs Developmental Competence After Fertilization

**FIGURE 3:**
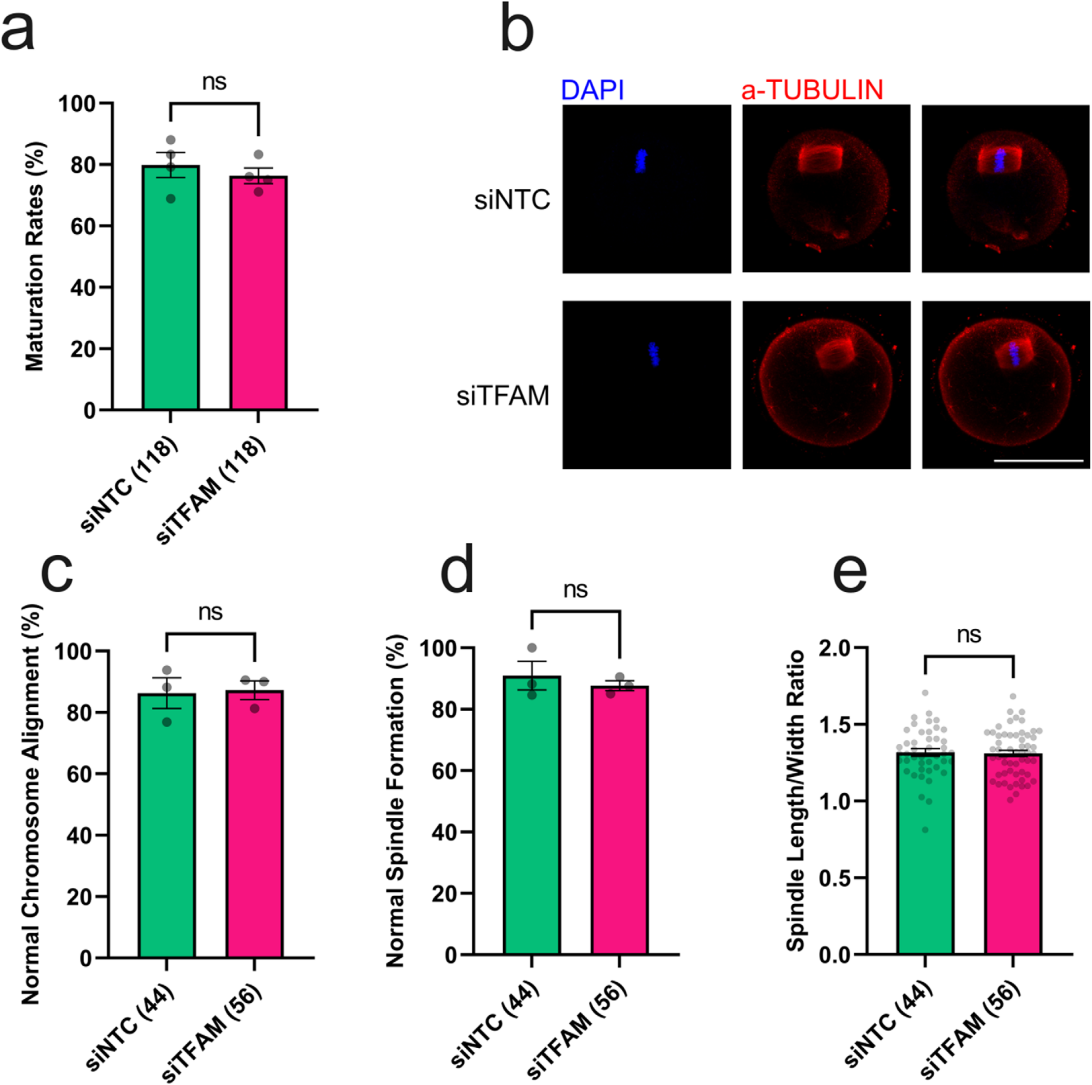
TFAM deficiency does not affect meiotic competence of IVG oocytes. (a) Percentage of oocytes reaching the metaphase II (MII) stage after in vitro maturation (IVM) (b) Representative confocal images showing spindle and chromosome morphology in control and TFAM knockdown oocytes. (c) Proportion of oocytes with normal spindle morphology. (d) Proportion of oocytes with normal chromosomal alignment. (e) Spindle length‐to‐width ratio in control and TFAM knockdown oocytes. Data represent mean ± SEM from three independent experiments. Asterisks indicate statistical significance (*p < 0.05; **p < 0.01; ***p < 0.001). Scale bar, 50 μm. siNTC, non‐targeting siRNA; siTFAM, *Tfam*‐targeting siRNA.

Given that mitochondrial function is a critical determinant of developmental competence, we next examined the effects of TFAM depletion on oocyte maturation and early embryonic development. Following IVG, oocyte–cumulus complexes were subjected to IVM, IVF, and in vitro embryo culture. The maturation rate and meiotic progression of TFAM knockdown oocytes were comparable to that of controls (Figure 3a, Table S2). Consistently, spindle morphology and chromosomal alignment at the MII stage appeared normal in both groups (Figure 3b–e), indicating that TFAM depletion does not disrupt meiotic competence.

**FIGURE 4:**
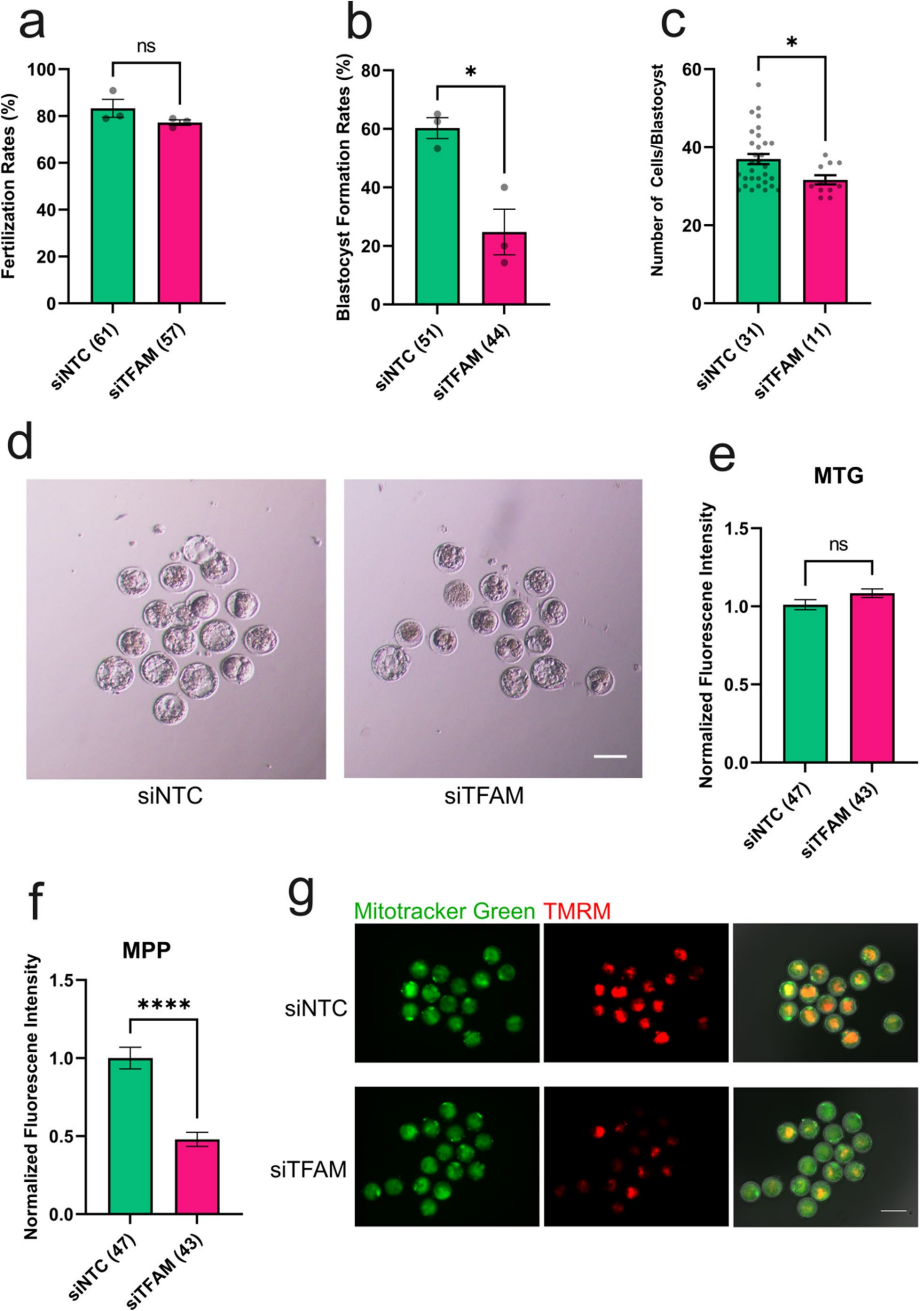
TFAM‐deficient IVG oocytes exhibit impaired post‐fertilization embryonic development. (a) Fertilization rates of control and TFAM knockdown oocytes. (b) Representative fluorescence images showing mitochondria distribution (MTG) and mitochondrial membrane potential (MMP) in control and TFAM knockdown 1‐cell embryos. (c) Normalized mitochondrial content levels indicated by MTG intensity. (d) Normalized mitochondrial membrane potential (MMP) levels. (e) Blastocyst formation rates. (f) Total cell numbers per blastocyst at Day 4.5. (g) Representative images of embryos at Day 4.5. Data represent mean ± SEM from three independent experiments. Asterisks indicate statistical significance (*p < 0.05). Scale bar, 100 μm. siNTC, non‐targeting siRNA; siTFAM, *Tfam*‐targeting siRNA.

Fertilization rates were also unaffected (Figure 4a). However, during preimplantation development, we observed a significant reduction in developmental progression beginning at the 4‐cell stage (Table S3). This was followed by a marked decrease in blastocyst formation, accompanied by a decrease in total cell number per blastocyst (Figure 4b–d). To further assess mitochondrial function in early embryos, we evaluated MMP and mitochondrial content in one‐cell embryos. Embryos were stained with the MMP‐dependent dye TMRM and the MMP‐independent mitochondrial marker MitoTracker Green, and TFAM knockdown embryos were compared with controls (Figure 4e–g). MMP was significantly reduced in TFAM knockdown embryos, whereas mitochondrial content and its distribution were unchanged, indicating that the reduced MMP reflects impaired mitochondrial function rather than a reduction in mitochondrial abundance.

Collectively, these findings demonstrate that although TFAM depletion does not affect oocyte maturation, fertilization, or mitochondrial content, it severely compromises post‐fertilization developmental competence by impairing MMP, underscoring the essential role of TFAM‐mediated mtDNA replication in establishing oocyte developmental competence.

## 4 Discussion

In this study, we characterized the dynamics of mtDNA accumulation during oogenesis, from early primary follicles to antral follicles. The relationship between mtDNA copy number and oocyte diameter was non‐linear, showing an accelerated increase during the later stages of oocyte growth. Notably, *Tfam* mRNA expression increased significantly, whereas Pink1 expression decreased at the secondary oocyte stage, suggesting that mtDNA replication is enhanced while mitophagy is suppressed. Together, these findings indicate a shift in mitochondrial homeostasis during oogenesis, in which biogenesis predominates over degradation to ensure sufficient mitochondrial content prior to fertilization [36].

TFAM proved essential for mtDNA replication during oogenesis. RNAi‐mediated *Tfam* knockdown suppressed mtDNA accumulation, confirming its critical role in mitochondrial biogenesis. Because oocyte mitochondria are metabolically quiescent with slow turnover [37], residual TFAM likely supported limited replication in knockdown oocytes. Although meiotic maturation, including polar body extrusion and spindle formation, was unaffected, TFAM knockdown resulted in a significant reduction in MMP. Given that mitochondrial content and distribution were unchanged, this functional impairment is most likely attributable to reduced mtDNA copy number per mitochondrion.

Although the molecular mechanisms linking mtDNA copy number to mitochondrial function in oocytes are not yet fully understood, impaired oxidative phosphorylation represents a plausible explanation for the reduced MMP observed in TFAM knockdown oocytes. mtDNA encodes several essential subunits of the mitochondrial respiratory chain complexes, and mtDNA abundance is tightly coupled to cellular metabolic demands [38]. Consistent with this notion, previous studies using TFAM knockdown models demonstrated that TFAM deficiency leads to mtDNA depletion and severe impairment of oxidative phosphorylation [39]. Nevertheless, because respiratory chain complexes consist of nearly 90 subunits encoded by both mitochondrial and nuclear genomes, elucidating the precise mechanisms underlying oxidative phosphorylation defects in oocytes will require comprehensive transcriptomic and proteomic analyses. Understanding how reductions in mtDNA copy number compromise mitochondrial function in mammalian oocytes therefore remains an important subject for future investigation.

Reduced mtDNA copy number has been associated with age‐related declines in oocyte quality [40] and reproductive pathologies such as ovarian insufficiency [41]. In contrast, conditional *Tfam* knockout models have shown that oocytes with severely reduced mtDNA can still reach the blastocyst stage [18, 42], likely due to compensatory support from the in vivo follicular environment. While preimplantation embryos can tolerate relatively low mtDNA copy numbers, post‐implantation development requires mtDNA levels above a critical threshold that is normally supported by the intact in vivo follicular environment. Oocytes rely heavily on balanced lipid and glucose metabolism for energy production, which is supported by the follicular environment and supplied through surrounding cumulus cells [43]. Bidirectional communication between the oocyte and cumulus and granulosa cells—mediated by gap junctions [19, 20], metabolic coupling [21], and paracrine signaling [22, 23] —is robust in vivo but is not fully reconstituted under IVG conditions [24]. Consequently, IVG culture systems lack sufficient metabolic buffering capacity, rendering mitochondrial dysfunction a limiting factor at earlier developmental stages. In this context, the critical mtDNA threshold required to sustain normal embryonic development in IVG‐derived oocytes is likely higher than that reported for in vivo–grown oocytes (approximately 40 000–50 000 copies per oocyte) [18]. TFAM deficiency may further exacerbate these mitochondrial abnormalities, suggesting that insufficient TFAM expression, together with mtDNA depletion, contributes to mitochondrial dysfunction in IVG oocytes. These findings are also consistent with the concept that oocyte maturation comprises both nuclear and cytoplasmic maturation. While nuclear maturation can proceed normally, insufficient cytoplasmic maturation—particularly under in vitro maturation or growth conditions—can compromise developmental competence [44, 45]. The reduction in mtDNA copy number and accompanying decrease in MMP observed following TFAM knockdown likely reflect impaired cytoplasmic maturation, ultimately leading to diminished embryonic development after fertilization.

Oocytes generated in IVG systems exhibit abnormal mitochondrial distribution, reduced mtDNA content, and impaired metabolic function [28, 46], all of which contribute to reduced developmental competence. In the present study, control IVG oocytes displayed a wide range of mtDNA copy numbers, with a mean value of approximately 120,000 copies per oocyte. By contrast, previous studies using droplet digital PCR—a highly sensitive and accurate quantification method—reported that fully grown oocytes from sexually mature mice contain approximately 140 000–150 000 mtDNA copies per oocyte [47]. This discrepancy further underscores the suboptimal quality of IVG oocytes and may partially explain the phenotypes observed in this study. Optimizing IVG conditions to better mimic physiological mitochondrial biogenesis may therefore improve oocyte quality and developmental outcomes. Given that TFAM knockdown during IVG reduces mtDNA content, it is tempting to speculate that supplementation with exogenous TFAM could restore mtDNA copy number and rescue IVG‐associated defects. However, previous studies have shown that excessive TFAM expression paradoxically reduces mtDNA levels [14, 17]. Overabundant TFAM promotes excessive nucleoid compaction, which suppresses mtDNA replication and transcription. Thus, precise fine‐tuning of TFAM expression to restore physiological TFAM‐to‐mtDNA ratios remains technically challenging. Future strategies aimed at enhancing endogenous TFAM expression or modulating upstream regulators of mitochondrial biogenesis may provide alternative approaches to improving IVG culture systems.

In summary, mtDNA copy number increases non‐linearly during oocyte growth, driven by key regulators of mitochondrial biogenesis such as TFAM. TFAM deficiency compromises mtDNA replication, mitochondrial function, and subsequent embryonic development, highlighting inadequate mitochondrial biogenesis as a potential bottleneck in in vitro folliculogenesis. Future studies should elucidate the molecular mechanisms coordinating mtDNA replication and mitochondrial quality control during oogenesis and develop culture systems that promote mitochondrial functionality to improve IVG‐derived oocyte competence.

## Funding

This work was supported by Japan Society for the Promotion of Science, 20K06627 and 23K08869.

## Ethics Statement

All institutional and national guidelines for the care and use of laboratory animals were followed.

## Conflicts of Interest

The authors declare no conflicts of interest.

## Supporting information


**Table S1:** Primer sequences used for PCR in this study.
**Table S2:** Effects of TFAM KD on maturation of IVG‐derived mouse oocytes.
**Table S3:** Effects of TFAM KD on fertilization and early embryonic development of IVG‐derived mouse oocytes.
**Figure S1:** Knockdown efficiency of TFAM siRNA measured by qPCR.

## Data Availability

The data that support the findings of this study are available from the corresponding author upon reasonable request.
