# Supplementary material for: TFAM‐Mediated mtDNA Replication is Essential for Developmental Competence of In Vitro Grown Oocytes
Source: Reprod Med Biol. 2026 Feb 23;25(1):e70031. doi: 10.1002/rmb2.70031 (PMC12928050; doi:10.1002/rmb2.70031)
Supplement: Supplementary file 1 — Table S1: Primer sequences used for PCR in this study. Table S2: Effects of TFAM KD on maturation of IVG‐derived mouse oocytes. Table S3: Effects of TFAM KD on fertilization and early embryonic development of IVG‐derived mouse oocytes. Figure S1: Knockdown efficiency of TFAM siRNA measured by qPCR. [file RMB2-25-e70031-s001.docx]

Table S1: Primer sequences used for PCR in this study.

| ***Gene*** | **Sequences** | **Reference** | **Product Size (bp)** |
| --- | --- | --- | --- |
| *ND1* | FP: CGATTAAAGTCCTACGTGATCTGA  RP: CTGGGAGAAATCGTAAATAGATAGAAA | NC_005089.1 | 80 |
| *Tfam* | FP: CATTTATGTATCTGAAAGCTTCC  RP: CTCTTCCCAAGACTTCATTTC | NM_009360 | 171 |
| *siTFAM* | FP: TCAGTCTGGGAAGGGAATGGG  RP: ATGCCTTCAGCTCCTCCCAAC | NM_009360 | 209 |
| *Polg* | FP: CCTAAGCTCATGGCACTGAC  RP: TGCTGCTTCCCCTGTTCAAG | NM_017462 | 202 |
| *Polg2* | FP: ACAGCAATCAGACACCCAG  RP: TTCTATTGGCTCCTTTCCCC | NM_015810 | 245 |
| *Nrf1* | FP: TCTCACCCTCCAAACCCAAC  RP: CCCGACCTGTGGAATAACTTG | NM_010938 | 254 |
| *Nrf2* | FP: GTCTTCACTGCCCCTCATC  RP: TCGGGAATGGAAAATAGCTCC | NM_010902.5 | 122 |
| *Sirt1* | FP: CTCTGAAAGTGAGACCAGTAGC  RP: TGTAGATGAGGCAAAGGTTCC | NM_001159589.2 | 94 |
| *Twinkle* | FP: GCTGGAAGAGCAACTGGACAAG  RP: GTGTCTATGACGGACCTGATGC | NM_001348259.1 | 111 |
| *Pink1* | FP: GCTTGCCAATCCCTTCTATGGC  RP: TAAGCGTGCAGACGGTCTCTTG | NM_026880.2 | 163 |
| *Parkin* | FP: GCTTGACACGAGTGGACCTGAG  RP: AGGTGGGTTTAACTGCTGGACC | NM_001317726.2 | 126 |
| *Ppia* | FP: CGCGTCTCCTTCGAGCTGTTTG  RP: TGTAAAGTCACCACCCTGGCACAT | NM_008907 | 150 |
| *H2afz* | FP: ACAGCGCAGCCATCCTGGAGTA  RP: TTCCCGATCAGCGATTTGTGGA | NM_016750 | 202 |
| *Actb* | FP: CATTGCTGACAGGATGCAGAAGG  RP: TGCTGGAAGGTGGACAGTGAGG | NM_001313923.1 | 138 |

Table S2: Effects of TFAM KD on maturation of IVG-derived mouse oocytes.

|  | No. (Ratio) of oocytes at GV stage | No. (Ratio) of oocytes at GVBD stage | No. (Ratio) of oocytes at MI stage | No. (Ratio) of oocytes at MII stage |
| --- | --- | --- | --- | --- |
| **siNTC** | 9/118 | 4/118 | 13/118 | 92/118 |
|  | 7.4 ± 1.1 | 4.1 ± 1.7 | 8.6 ± 4.5 | 79.8 ± 4.1 |
| **siTFAM** | 12/118 | 3/118 | 14/118 | 89/118 |
|  | 9.5 ± 3.2 | 3.0 ± 3.0 | 11.1 ± 1.8 | 76.4 ± 2.6 |

Data represent mean ± SEM from four independent experiments. No statistical significance was found in this data set.

Table S3: Effects of TFAM KD on fertilization and early embryonic development of IVG-derived mouse oocytes.

| Total | No. (Ratio) of fertilized oocytes | No. (Ratio) of 2-cell embryos | No. (Ratio) of 4-cell embryos | No. (Ratio) of morula stage embryos | No. (Ratio) of blastocysts | No. of blastocyst cell |
| --- | --- | --- | --- | --- | --- | --- |
| **siNTC** | 51/61 | 45/51 | 38/51 | 35/51 | 31/51 | 37.0 ± 1.3 |
|  | 83.3 ± 3.8 | 86.7 ± 4.0 | 74.4 ± 0.6 | 68.1 ± 3.7 | 60.3 ± 3.5 |  |
| **siTFAM** | 44/57 | 32/44 | 17/44 | 12/14 | 11/44 | 32.2 ± 1.4* |
|  | 77.2 ± 1.2 | 72.6 ± 7.1 | 38.4 ± 5.3** | 27.0 ± 7.4** | 24.8 ± 7.8* |  |

Data represent mean ± SEM from three independent experiments. Within a column, asterisks indicate statistical significance (P* < 0.05, P** < 0.01).


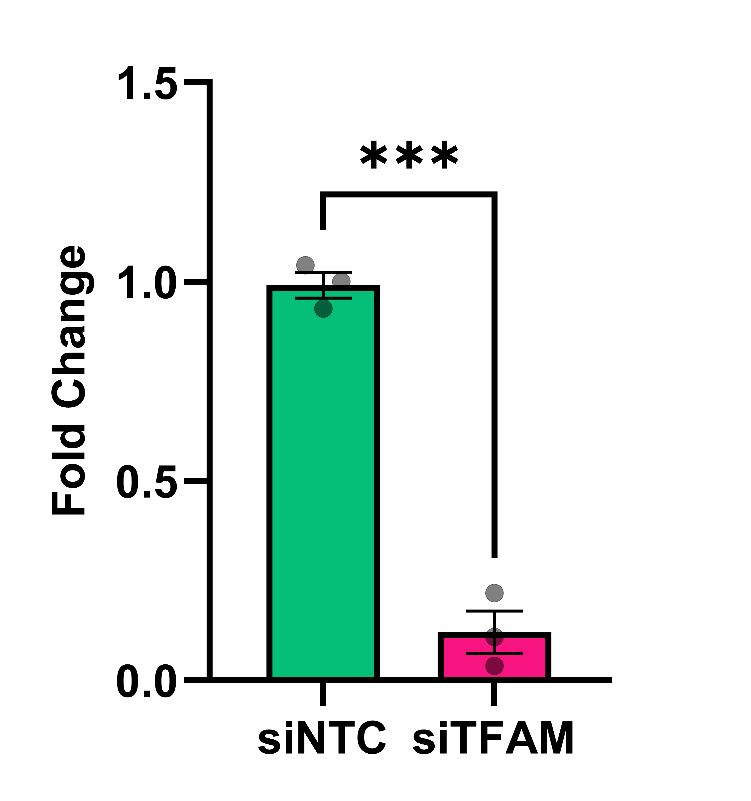


Figure S1: Knockdown efficiency of TFAM siRNA measured by qPCR.

Primers flanking the siRNA binding site were used to analyze the mRNA abundance. Data represent mean ± SEM from three independent experiments. Asterisks indicate statistical significance (P*** < 0.001)
